# Synchronous or sequential cementless bilateral total hip arthroplasty for osseous ankylosed hips with ankylosing spondylitis

**DOI:** 10.1186/s12891-021-04142-7

**Published:** 2021-03-24

**Authors:** Ping Mou, Wei Nan Zeng, Yu Chen, Zongke Zhou

**Affiliations:** 1 Department of Orthopedics, West China Hospital, Sichuan University, #37 Guoxue Road, 610041 Chengdu, People’s Republic of China; 2 Department of Orthopedics, Chongqing General Hospital, University of Chinese Academy of Sciences, 400014 Chongqing, China; 3 Clinical medicine, West China Medical School, West China Hospital, Sichuan University, 610041 Chengdu, Sichuan Province People’s Republic of China

**Keywords:** Bilateral total hip arthroplasty, Ankylosing spondylitis, Ankylosed hips

## Abstract

**Background:**

Bilateral osseous ankylosed hips secondary to ankylosis spondylitis (AS) are relatively rare but impact the quality of life hugely. Cementless total hip arthroplasty (THA) for bilateral osseous ankylosed hips with AS is a challenging procedure. No previous literature compares the clinical outcomes of synchronous and sequential bilateral THA for these special patients.

**Methods:**

23 patients (46 hips) were retrospectively analyzed and divided into bilateral THA synchronously (group A) and sequentially (group B). The clinical measurement, radiological assessments, and complications were compared. Independent sample T test was used for data analysis.

**Results:**

Harris Hip Scores (HHS) improved greatly for both groups (*P* = 0.58) as well as the range of motion (*P* = 0.64). But group B can realize shorter time (3.6 ± 1.2 days) to walk for the first time postoperatively (*P* = 0.02). Group A needed more blood transfusions (*P* = 0.028). For group A, no statistical difference was found in the bilateral inclination of cup (IC) (*P* = 0.48) and femoral offset (FO) (*P* = 0.07). For group B, no statistical difference was observed in bilateral IC (*P* = 0.37) but in bilateral FO (*P* = 0.04). Group A showed the fewer difference of bilateral IC (*P* = 0.02), while comparative measurements were found for two groups in the difference of bilateral FO (*P* = 0.78) and leg length discrepancy (*P* = 0.83). For both groups, the total hospital expense for each patient was similar and almost all patients were very satisfied with the outcomes. For group A, one patient encountered femoral fracture intraoperatively and another patient encountered hip dislocation and delay union of wound. 3 hips from group A and 3 hips from group B encountered heterotopic ossification.

**Conclusions:**

Our retrospective research demonstrated that cementless bilateral THA was a reliable treatment for osseous ankylosed hip due to AS. Synchronous and sequential bilateral THA can realize similarly satisfactory clinical outcomes and radiographic evaluation.

## Background

Ankylosing spondylitis (AS) is an inflammation spondyloarthritis affecting the axial spine and peripheral joints and characterized by low back pain and limited range of motion (ROM) of lumbar spine [1, 2]. And AS is diagnosed using the modified New York criteria requiring image change of sacroiliitis and painful reduction of lumbar spine ROM as well as stiffness more than 3 months [3]. Hips are the most common peripheral joints involved and approximately 25 %~50 % of patients can encounter hip involved [4, 5], of which 90 % presents bilateral hip ankylosis [6]. The end-stage hip ankylosis usually manifests osseous ankylosis with the total loss of hip ROM [7]. Although bilateral hip fusion leads to stable and painless hip, yet the loss of hip function and premature degeneration of neighboring joints harm the quality of life, especially for suboptimal hip fusion for a long period [8–10]. Total hip arthroplasty (THA) can relieve pain and recover the ROM of the hip to improve joint function and self-care ability [11]. Also, many literatures have reported good radiographic outcomes and improvements in hip function [7, 12–14]. However, for bilateral osseous ankylosed hips with AS, there is no consensus on synchronous or sequential THA for these special patients.

There are many difficulties for bony ankylosed hip conversion to THA, which include the exposure of surgical area [7], the ambiguous identification of original joint plane [7], disuse osteoporosis [15], weakness of abductor muscle [16], and pelvic obliquity [12]. For bilateral bony ankylosed hips with AS, while synchronous THA may prolong operation time and cause more blood loss, bilateral hip lesions can be solved simultaneously. Moreover, two flectional hips can be favorable for functional rehabilitation postoperatively. Comparatively, sequential THA for these patients shorten operation time and cause less surgical damage, yet the temporary unhandled ankylosed hip can be an obstacle to the rehabilitation of the operated hip. Additionally, the total two hospitalization expenses may be more than that of synchronous procedure. Currently, the literatures just reported synchronous or sequential THA for osseous ankylosed hips with AS [7, 12–14] with the limitations such as different types of cementless cup, cemented or cementless stem [12], short time of follow-up [14], and small study population [7]. There was no report comparing the clinical and radiographic outcomes of synchronous and sequential cementless bilateral THA for osseous ankylosed hips with AS.

To our knowledge, this study compared the clinical and radiographic outcomes of synchronous and sequential cementless bilateral THA to correct hip osseous ankylosis with AS for the first time. And it was also the currently largest sample-size research on the outcomes of THA for bilateral ankylosed hips with AS. It was hypothesized that for osseous ankylosed hips with AS, synchronous cementless bilateral THA can realize similar outcomes with sequential THA.

## Methods

Data were collected by retrospective review of a prospective database from January 2010 to December 2017. Study approval was obtained from the Clinical Trials and Biomedical Ethics Committee of West China Hospital, and all participants signed informed consents for the use of their data. The data included patient demographics, time of walking for the first time postoperatively, preoperative hip flexion contracture, total hospital expense, satisfactory level, range of flection-extension motion, Harris Hip Scores (HHS), transfusion, radiological assessments, and complications.

### Patients

The inclusion criteria: patients diagnosed with AS and performed primary cementless THA; patients with bilateral osseous ankylosed hips and trabecula bridging the joint plane on radiograph; total loss of hip ROM; patients receiving bilateral THA synchronously or sequentially. the exclusion criteria: bilateral osseous ankylosed hips caused by other reasons; unilateral osseous ankylosed hip with AS; fibrous ankylosis of the hip and no trabecula bridging the joint plane on radiograph; patients just receiving unilateral THA; the patients lost to follow-up.

Ultimately, we analyzed the data of the patients receiving bilateral THA synchronously (group A) and bilateral THA sequentially (group B). The synchronous or sequential procedures were based on patients’ financial situation and patients’ choice. For the surgeon, surgery could be performed, if the patients meet preoperative screening. All hips were identified as bony ankylosed hip with the total loss of ROM. The patients have been diagnosed with AS for many years and irregular treatment was done. All patients had total loss ROM of lumbar without ankylosis of the knee. 1 patient of group A and 1 patient of group B received lumbar spinal osteotomy and fusion before THA. We called up the patients to accomplish follow-up. And we obtained the latest clinical and radiological outcomes. 11 patients (22 hips) in group A and 12 patients (24 hips) in group B were followed up. The demographic data of the patients were summarized in Table 1. The mean duration of the follow-up was 81.9 ± 36.3 months for group A and 79.9 ± 29.1 months for group B (*P* = 0.84).

**Table 1:** Baseline Characteristics of all included patients

Variable	Group A (22 hips)	Group B (24 hips)	T value	P value
M/F	10/1	10/2	-	-
Height (cm)	161.9 ± 9.0	160.0 ± 5.1	0.88	0.37
Weight (Kg)	59.9 ± 10.7	58.1 ± 6.6	0.69	0.50
BMI (Kg/m^2^)	22.7 ± 3.3	22.7 ± 2.5	-0.02	0.98
Friction couples				
Ceramic-on ceramic	18	18	-	-
Ceramic-on-polyethylene	4	6	-	-
Additional screws fixation (patients/hips)	5/10	6/12	-	-
Average follow-up time (months)	81.9 ± 36.3	79.9 ± 29.1	0.21	0.84

Group A: synchronous cementless bilateral total hip arthroplasty

Group B: sequential cementless bilateral total hip arthroplasty

*BMI* body mass index

### Surgical procedures

All operations were performed by a group of surgeons specializing in THA. Considering the different degrees of hip deformities, the worse hip was done first. After general anesthesia, all patients were positioned in the lateral position and exposed to the hips through a posterolateral approach. The femoral neck was identified according to the lesser trochanter and osteotomy was done without hip dislocation. For the hip with external rotation deformity, osteotomy behind the femoral neck was difficult. So, we usually performed an osteotomy in the front of the femoral neck to avoid damage of the greater trochanter and posterior acetabulum. No trochanteric osteotomy was performed. Reamers with the gradual increase in diameter were used to prepare the acetabulum in the medial direction and the counterrotation technique was used to avoid over-reaming of the osteoporotic acetabulum. According to foveal soft tissue and incomplete gray ossifying cartilage, we located the original joint plane. The optimal cup size and cup inclination of the acetabulum implant were identified by intraoperative fluoroscopy. And the anteversion of the cup was confirmed with the indication of transverse acetabular ligament or long axis of the body. If the initial press-fit was not satisfactory, additional screws would be used to fix the cup before inserting the liner.

For the preparation of the femoral canal, sequentially larger reamers were used to enlarge the canal until the diaphyseal cortex was involved. The lesser trochanter and ipsilateral transcondylar line indicated the anteversion of the stem. Then femoral trial prosthesis was inserted to correct the leg length discrepancy (LLD), check the stability in all directions, and optimize the femoral offset. At last, the cementless femoral prosthesis and femoral head were inserted. We checked again the stability and ROM of the hip to ensure optimal angles and postoperative mobility. If the hip can’t be abducted more than 15° passively, we would cut off the adductor tendon. At the end of the procedure, the external rotator muscles were restored and the drainage was selected according to the time of operation and blood loss before incision suturing. Only one single brand (DePuy, Warsaw, IN) of the cementless cup and stem implants were used for all patients. The friction interfaces used included ceramic-on-ceramic (CoC) or ceramic-on-polyethylene (CoP), which were decided according to the age, level of activity, and patients’ financial situation.

### Perioperative Regimen

Isometric exercises and positive motion exercises were conducted in bed after recovering from anesthesia. Prophylactic intravenous antibiotics were used within the first 24 h postoperatively. Additionally, low-molecular-weight heparin (LMWH) was systematically managed to prevent deep venous thrombosis (DVT). A half-dose (2000 IU in 0.2 mL) of LMWH was initially administered subcutaneously 6 h postoperatively and a full dose (4000 IU in 0.4 mL) was repeated at 24-hour intervals subsequently until hospital discharge. After discharge, all patients routinely received 10 mg rivaroxaban for 15 days. And non-steroidal anti-inflammatory drugs (NSAIDs) were used to relieve pain and reduce the chance of heterotopic ossification (HO) for two weeks. For unbearable postoperative pain, additional painkillers by intravenous or intramuscular injection were added. The drainage tube was removed within 24 h.

From the first postoperative day on, the patients were allowed to partial weight-bearing exercises with the help of the walker aid, then exercise with the help of cane after 2 weeks and full weight-bearing exercises after 4 weeks without help. Moreover, for the patient with hip flection deformity preoperatively, the hip gradually extended under the circumstances of bearable pain. Routine clinical follow-up visits were conducted at 2 weeks, 4 weeks, 12 weeks, and 6 months after surgery and annually.

### Clinical measurements

At the latest follow-up, clinical details were recorded including hip flection-extension ROM and Harris Hip Scores (HHS) of the two groups [17]. A special ruler was used to measure the range of hip flexion and extension when the patients were in the supine position. ROM and Harris scores were examined by 2 authors to reduce the bias. Satisfactory levels were divided into very satisfactory, satisfactory, unsatisfactory, and very unsatisfactory. Additionally, from our database, we collected the data such as the time of walking for the first time postoperatively, preoperative hip flexion contracture, total hospital expense for each patient, the average blood transfusions, and the blood transfusion rate. The standard of blood transfusions referred to the guidelines of the National Ministry of Health, recommending blood transfusion for hemoglobin level less than 7 g/dL until the level reached or exceeded 8 g/dL. Additionally, when hemoglobin level fluctuated between 7 and 10 g/dL, transfusion would be considered in patients with symptomatic anemia (severe mental status changes, palpitations, and/or pallor).

### Radiological assessments

Standard anteroposterior radiographs were obtained preoperatively, immediately after surgery, and at the latest follow-up. And the radiological data were collected and analyzed by the same two authors. The assessments included the inclination of the cup (IC), the difference of bilateral IC, the femoral offset (FO), the difference of bilateral FO and LLD at the latest follow-up. IC was measured directly on the AP radiograph. The angle crossed by the horizontal line connecting both teardrops and the line through the longest diameter of the elliptical opening of the acetabular cup rim was recorded and regarded as IC [12]. If the teardrops were unrecognizable or the pelvis was asymmetric, we firstly bisected the sacrum with a vertical line A and secondly drawn a perpendicular line B to line A [12]. So, line B can be used as a horizontal line. FO was defined as the vertical distance from the center of the femoral head to the ipsilateral anatomical femoral axis [18]. LLD was assessed by the standardized-trochanteric method to avoid the influence of pelvic obliquity and femoral inclination on the radiographs [19]. The standardized-trochanteric method requires the vertical distance from the inter-teardrop line to the center of rotation and the femoral vertical distance (center of rotation to the lesser trochanter) reference to the femoral anatomical axis. So, the unilateral distance is defined as the difference between the two vertical distances. And LLD is equal to the difference between the two unilateral distances.

### Complications

The complications were recorded and evaluated including early-onset and late-onset complications during the perioperative period and at the latest follow-up. The early-onset complications consisted of dislocation, wound complication, infection, intraoperative fracture, DVT, pulmonary embolism, and nerve palsy. The data were collected from the database. Meanwhile, the late-onset complications consisted of postoperative dislocation, HO, osteolysis, and aseptic loosening at the latest follow-up, which were assessed by the same two authors. Based on Brooker classification [20], we analyzed and classified the degree of HO. Osteolysis was defined as cystic or scalloped lesions with a diameter of more than 2 mm on radiograph [21, 22]. According to the criteria of DeLee et al. [23], the acetabular component was considered loose if a complete radiolucent line thicker than 1mm at bone-implant interface or migration of the component showed. Besides, the femoral implant stability was evaluated according to Engh et al. [24], the stem was considered loose if subsidence more than 2 mm or angular shift of the stem more than 2° showed.

### Statistical analysis

Statistical analysis was performed using SPSS software for Windows Version 22.0 (SPSS, Chicago, IL). The level of statistical significance was set at p<0.05. The results were expressed as the mean ± standard deviation. Independent sample T test was used for data analysis.

## Results

### Clinical outcomes

The preoperative hip flexion contracture showed (39.3 ± 20.3)° for group A and (37.7 ± 18.7) ° for group B (*P* = 0.40) in Table 2. The average preoperative HHS increased from preoperative 30.5 ± 5.9 to 84.0 ± 2.8 at the latest follow-up for group A and from preoperative 31.4 ± 4.6 to 83.4 ± 2.0 for group B. Moreover, all of the hips lost total ROM of hip preoperatively, but at the latest follow-up, the average flection-extension ROM was 85.7 ± 4.5° for group A and 85.1 ± 4.1° for group B. The average flexion and extension were 86.5 ± 4.4° and 0.73 ± 2.4° for group A and 85.7 ± 3.5° and 0.54 ± 1.9° for group B, respectively. No statistical difference was found in both groups, but large improvement was realized postoperatively (Table 2).

**Table 2:** Clinical outcomes of all included patients preoperatively and postoperatively

Variable	Group A (22 hips)	Group B (24 hips)	T value	*P* value
Preoperative hip contracture (°)	39.3 ± 20.3	37.7 ± 18.7	0.28	0.40
Preoperative HHS	30.5 ± 5.9	31.4 ± 4.6	-0.62	0.58
Postoperative HHS	84.0 ± 2.8	83.4 ± 2.0	0.89	0.38
Postoperative extension (°)	0.73 ± 2.4	0.54 ± 1.9	0.29	0.77
Postoperative flexion (°)	86.5 ± 4.4	85.7 ± 3.5	0.67	0.50
Postoperative ROM (°)	85.7 ± 4.5	85.1 ± 4.1	0.47	0.64
Time of walking for the first time postoperatively	5.1 ± 2.6	3.6 ± 1.2	2.43	0.02*
average blood transfusions (U)	3 ± 3.97	0.71 ± 1.99	2.29	0.028*
blood transfusion rate	8/11(72.7 %)	5/24 (20.8 %)	-	-

Group A: synchronous cementless bilateral total hip arthroplasty

Group B: sequential cementless bilateral total hip arthroplasty

*ROM* range of motion

*HHS* Harris Hip score

*P* values with statistical significance are marked with *

However, the statistical difference was found that the patients receiving synchronous bilateral THAs needed more time to walk for the first time postoperatively with 5.1 ± 2.6 days, while it was 3.6 ± 1.2 days for group B (*P* = 0.02) (Table 2). The average interval time between two operations was 40.8 ± 23.0 days for group B. Furthermore, blood transfusions perioperatively were also different between the two groups. Average blood transfusions were 3 ± 3.97 U for group A and 0.71 ± 1.99 U for group B (i = 0.028), while the blood transfusion rate was 8/11 (72.7 %) for group A and 5/24 (20.8 %) for group B. The average hospital expense of bilateral THA was (108,660 ± 6440) RMB for group A, while it was (113,238 ± 6759) RMB for group B. Although no statistical difference was found between groups on hospital expense (*P* = 0.90), the average hospital expense for group A was less than that of group B. For group A, 10 patients were very satisfactory and 1 patient was satisfied with the outcomes. And all 12 patients of group B were very satisfied with the outcomes. None was unsatisfactory with the outcomes.

### Radiographic evaluation

**Fig. 1:**
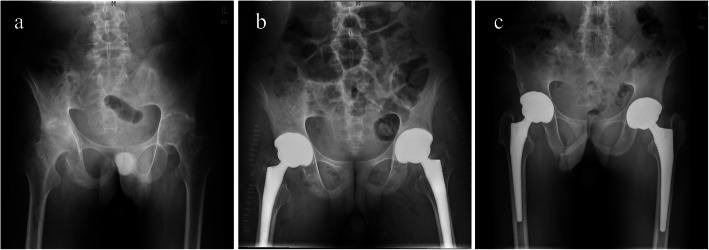
Case presentation of synchronous cementless bilateral total hip arthroplasty for osseous ankylosed hips with ankylosing spondylitis **a**: A man was diagnosed with ankylosing spondylitis and showed bilateral osseous ankylosed hips preoperatively. **b**: The radiograph of the pelvis after surgery immediately showed similar inclination of cup and femoral off-set as well as small leg-length discrepancy **c**: The radiograph of the pelvis at 100-month follow-up showed no aseptic loosening and migration of the component

**Fig. 2:**
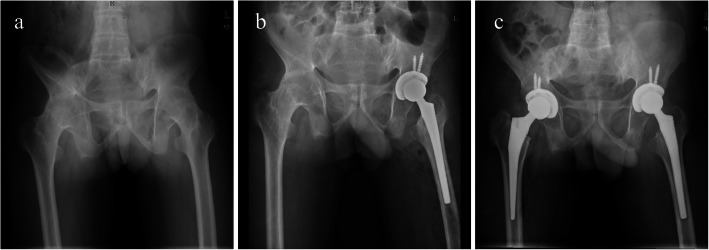
Case presentation of sequential cementless bilateral total hip arthroplasty for osseous ankylosed hips with ankylosing spondylitis. **a**: A man was diagnosed with ankylosing spondylitis and showed bilateral osseous ankylosed hips preoperatively. **b**: The radiograph of the pelvis before contralateral THA showed excellent prosthesis position and size of firstly performed THA. **c**: The film of the pelvis at 97-month follow-up showed superior radiological parameter and good fixation of the prosthesis

For group A, at the latest follow-up, there was no difference in the average IC between the right and left hips (*P* = 0.48) (Fig. 1). Similarly, the same result was found in group B (*P* = 0.37) (Fig. 2). For the FO, the patients in group A showed no difference between the right and left hips (*P* = 0.07), while the statistical difference was found in group B (*P* = 0.04) (Table 3). Also, we compared the difference of bilateral IC and the difference of bilateral FO for both groups (Table 4). The differences of bilateral IC were 3.0 ± 2.1°for group A and 5.5 ± 2.4°for group B (*P* = 0.02). Meanwhile, the differences of bilateral FO were 0.35 ± 0.27 cm for group A and 0.32 ± 0.21 cm for group B. And LLD were 0.48 ± 0.39 cm for group A and 0.45 ± 0.31 cm for group B. No differences were found between groups for the differences of bilateral FO (*P* = 0.78) and LLD (*P* = 0.83). 5 patients (10 hips) for group A and 6 patients (12 hips) for group B used additional screws for fixation (Table 1) and no influence on the final results was observed.

**Table 3:** Radiographic evaluation of the included patients between groups

Variable	Group A (22 hips)				Group B (24 hips)			
	Right hips	Left hips	T	P	Right hips	Left hips	T	P
Average IC (°)	39.6 ± 3.8	38.8 ± 5.1	0.73	0.48	41.5 ± 4.4	39.9 ± 5.2	0.94	0.37
Average FO (cm)	4.6 ± 0.6	4.4 ± 0.6	2.16	0.07	4.1 ± 0.3	3.9 ± 0.3	2.34	0.04*

Group A: synchronous cementless bilateral total hip arthroplasty

Group B: sequential cementless bilateral total hip arthroplasty

*IC* inclination of cup; *FO* femoral offset

*P* values with statistical significance are marked with *

**Table 4:** Radiographic evaluation of the included patients between groups postoperatively

Variable	Group A (22 hips)	Group B (24 hips)	T value	*P* value
The difference of bilateral IC (°)	3.0 ± 2.1	5.5 ± 2.4	-2.63	0.02*
The difference of bilateral FO (cm)	0.35 ± 0.27	0.32 ± 0.21	0.28	0.78
LLD (cm)	0.48 ± 0.39	0.45 ± 0.31	0.22	0.83

Group A: synchronous cementless bilateral total hip arthroplasty

Group B: sequential cementless bilateral total hip arthroplasty

*IC* inclination of cup; *FO* femoral offset; *LLD* leg length discrepancy

*P* values with statistical significance are marked with *

### Complications

Only two hips in group A encountered early-onset complications. One femoral encountered fracture intraoperatively, which was fixed with several double-loop cerclage wires immediately. The patients mainly conducted the functional exercise in bed until the fracture healed. Another patient suffered from hip dislocation postoperatively and delay union of wound. With effective handling, no dislocation happened evermore and the wound recovered ultimately. For late-onset complications, three hips (13.6 %) in group A and three hips (12.5 %) in group B encountered asymptomatic HO, all of which belonged to Brooker I. Other complications such as dislocation, osteolysis, and loosening were not observed.

## Discussion

The most important finding of this study was that cementless bilateral THA for osseous ankylosed hips with AS showed good clinical outcomes and almost all patients were very satisfied with the functional improvements and no statistical difference was found on total hospital expense for both groups. Besides, compared to sequential bilateral THA, synchronous bilateral THA can realize the comparative clinical and radiographic outcomes with an average follow-up of more than 79 months. But synchronous bilateral THA may need more blood transfusions and more time to walk for the first time postoperatively.

When AS patients have presented bilateral osseous ankylosed hips, THA has been delayed for a long time. Hip fusion may avoid pain from joint motion but pain due to degeneration of adjacent joints may gradually appear and aggravate. And it is more technically difficult for osseous ankylosed hip performed THA than non-ankylosed hip [7]. Fortunately, efficient surgical skills about THA for ankylosed hip due to AS were recommended [7, 12, 15]. Also, the recent study has reported satisfactory long-term survivorship for THA to 89.4 % at 15 years, 70.2 % at 20 years, and 57.9 % at 25 years [25]. So, we suggest that for AS patients, once the hip present pain, obvious joint space stenosis, and noneffective conservative treatment, THA should be scheduled.

Several challenges may be faced when performing THA for the bony ankylosed hip. Firstly, there were three approaches to expose the coxa including posterolateral approach [7, 13, 14], posterior approach [26–28], and direct lateral approach with trochanteric osteotomy [6, 12]. And the direct lateral approach was usually performed with trochanteric osteotomy, which may result in possible nonunion of the greater trochanter [13] and nerve injury especially the superior gluteal nerve [29, 30]. The other two approaches can realize sufficient exposure to the acetabulum and proximal femur. But more attention should be attached to preventing the risk of sciatic nerve injury due to scarred and contracted soft tissues around the hip caused by AS [31, 32]. These two approaches both can achieve a safe and high-quality THA and we recommended them as effective approaches to perform THA. Secondly, it is difficult that how to conduct acetabular reaming appropriately and insert the cup with a suitable size for the bony ankylosed hip. It is helpful to identify the original joint plane and confirm the depth of reaming that the foveal soft tissue and incomplete gray ossifying cartilage in the interface of femoral head and acetabulum [7, 13, 14]. Of course, a detailed preoperative plan and intraoperative radiography facilitate judging the suitable size and avoiding over-reaming. Thirdly, the asymmetric pelvis caused a great challenge of cup insertion in the appropriate direction [12]. The malposition of the cup can lead to dislocation or impingement and accelerate liner wear [12, 33]. Although the study has reported different ways to modify the angle of the cup to realize the safe range (40°±10° for inclination and 15°±10°for anteversion) according to different kinds of deformities [34], yet we consider them complicated and unquantifiable. We suggest intraoperative radiography and tests of hip stability in all directions to avoid dislocation and impingement. The results of our study also demonstrated the validity of the methods recommended by us.

The purpose of THA for the bony ankylosed hip is to correct hip deformity and restore fundamental flexion-extension ROM from the fusion hip. It was reported that the increase of ROM and improvement in self-care ability were two important factors associating with patient satisfaction [11]. Although the conversion of the bony ankylosed hip to THA led to inferior ROM and HHS compared to primary THA (PTHA), THA for those patients can relieve pain, restore hip mobility, improve function, and correct LLD [13, 16]. However, THA for fusion hip can be more technically difficult, need more operation time, and cause more traumatic response compared to PTHA [7, 12, 14, 33], which may result in more blood loss and longer time of rehabilitation. Maybe it is more obvious for bilateral THA performed synchronously. The research has reported that the postoperative transfusion rate for one-stage bilateral PTHA was 29.2 % and unilateral PTHA was 15.9 % as well as more rehabilitation time required for bilateral PTHA [35]. And our study also showed more blood transfusions were needed for synchronous and sequential procedures. Furthermore, the synchronous demanded more transfusions and more time of rehabilitation than the sequential. Fortunately, with the extensive use of tranexamic acid in total joint arthroplasty [36, 37], the blood loss can be reduced obviously. Additionally, the total hospital expense for each patient of both groups was similar and almost all patients were very satisfied with the outcomes. The possible reason may be that THA restored the hip ROM and improved patients’ self-care ability as reported [11]. Also, this can account for the high satisfactory level of our study. Moreover, these two operation options can realize comparable HHS and flexion-extension ROM. So, for the patients with good nutritional status, one-stage bilateral THA may be an alternative means to cure bony ankylosed hip synchronously. And no difference was found in LLD, IC, and the difference of bilateral FO. Although the synchronous procedure can obtain less difference of bilateral IC, yet the average IC of both two procedures fluctuates between 30°and 50°. If we perform sample-size calculation, the estimate was based on the postoperative HHS among the 2 study groups using G*Power Version 3.1.9.2 (Franz Faul; Uni Kiel, Germany) software. The previous study of 31 hips who had undergone unilateral THA [28] and the previous study of 24 hips who had undergone synchronous bilateral THA [7] showed that the mean postoperative HHS were 87.1 ± 13.1 and 82.7 ± 6.9, respectively; to detect a treatment difference of 10 %, the sample size required for each group of the study was 90 hips. This sample size was calculated for independent samples T test assuming a standard effect size (d) = 0.42, an alpha level (two-tailed) = 0.05, and power = 0.8. The sample size was increased by 20 % to compensate for expected dropouts, resulting in 108 hips per group and a total number of 216 hips. The relatively small sample size (22 hips for group A and 24 hips for group B) may account for this difference.

The study reported that AS increased perioperative and postoperative complications after THA and high incidence of complications including wound complication, polyethylene wear, revision, and dislocation [38]. Survival analysis of THA for AS was 81.4 % at 15 years [6], which was lower than that of THA for other etiologies [25]. Also, the rate of polyethylene wear of THA for the bony ankylosed hip was high than that of PTHA concluded by Kim et al. [39]. Two reasons may explain the higher rates of implant failure for the bony ankylosed hip. Firstly, possible component malposition and abnormal spinopelvic mechanics contribute to abnormal stress to the implants and result in acceleration of polyethylene wear and increase of revision rate [40, 41]. Secondly, the patients receiving THA due to AS are younger and more active than average patients and present higher functional demand and physical activity [38]. According to our research, it was found that good survival of prosthesis for bony ankylosed hip on an average 79-months follow-up. Hip dislocation is another frequent complication. Possible component malposition and increased demand for the ROM of hip due to rigidity of spine added risk of hip dislocation when running daily activity [42]. Also, the weakness of abductor muscles was another risk of dislocation. Based on our research, appropriate prosthesis insertion lay the foundation for stability. Contracture of the soft tissue due to AS limited hip ROM and reduced the incidence of dislocation. And reasonable rehabilitation postoperatively was conducive to the strength of abductor muscles. Intraoperative periprosthetic fracture is another noticeable complication. Osteoporosis of the acetabulum and proximal femur is usually encountered for fusion hip due to long-term disuse [15]. When inserting the prosthesis, the excessive impact may cause cup protrusion into the pelvis or proximal femoral fracture resulting in poor primary fixation of the stem. So, appropriate impact and careful examination of possible fracture should be involved intraoperatively. If necessary, additional screws or the stem with distal fixation should be used also.

Several strengths and limitations were noticed in our study. First, the study was a retrospective evaluation of patients with a small sample size and short follow-up at a single center. But it was the currently biggest sample-size research about THA for bilateral bony ankylosed hips and the first research to compare clinical outcomes of the synchronous procedure with that of the sequential procedure. Second, although the surgery-specific information including intraoperative blood loss, total blood loss, and inflammatory biomarkers can’t be obtained, yet the total hospital expense for each patient and satisfactory level were reported for the first time. Third, Imaging changes of the pelvic and lumbar can affect postoperative dislocation of THA and stability of the prosthesis, but we just compared the clinical outcomes of the synchronous procedure with that of the sequential procedure as well as brief radiographic comparisons. And no detailed reports about imaging change were presented in the study. Our research team is studying the relationship of lumbar lesions caused by AS and stability of prosthesis as well as related imaging change. We will report our results in the future and we believe it can solve this problem.

## Conclusions

Our retrospective research demonstrated that cementless bilateral THA was a reliable treatment for osseous ankylosed hip due to AS. Synchronous and sequential bilateral THA can realize similarly satisfactory clinical outcomes and radiographic evaluation.

## Data Availability

Public access to the database is closed. For us, all related datasets were permitted to access and use by the Clinical Trials and Biomedical Ethics Committee of West China Hospital, Sichuan University. And the datasets used and/or analyzed during the current study are available from the corresponding author on reasonable request.

## Abbreviations

THA: Total hip arthroplasty
AS: Ankylosing spondylitis
HHS: Harris Hip Score
IC: Inclination of cup
FO: Femoral offset
ROM: Range of motion
LLD: Leg length discrepancy
CoC: Ceramic-on-ceramic
CoP: Ceramic-on-polyethylene
LMWH: Low-molecular-weight heparin
DVT: Deep venous thrombosis
NSAIDs: Non-steroidal Anti-inflammatory Drugs
HO: Heterotopic ossification

## References

[1] Braun J, Sieper J (2007). Ankylosing spondylitis. Lancet.

[2] Raychaudhuri SP, Deodhar A. The classification and diagnostic criteria of ankylosing spondylitis. Journal of autoimmunity. 2014;48–49:128 – 33. 10.1016/j.jaut.2014.01.015 24534717

[3] van der Linden S, Valkenburg HA, Cats A (1984). Evaluation of diagnostic criteria for ankylosing spondylitis. A proposal for modification of the New York criteria. Arthritis rheumatism.

[4] Tang WM, Chiu KY (2000). Primary total hip arthroplasty in patients with ankylosing spondylitis. J Arthroplast.

[5] Sochart DH, Porter ML (1997). Long-term results of total hip replacement in young patients who had ankylosing spondylitis. Eighteen to thirty-year results with survivorship analysis. The Journal of bone joint surgery American volume.

[6] Joshi AB, Markovic L, Hardinge K, Murphy JC (2002). Total hip arthroplasty in ankylosing spondylitis: an analysis of 181 hips. J Arthroplast.

[7] Bangjian H, Peijian T, Ju L (2012). Bilateral synchronous total hip arthroplasty for ankylosed hips. International orthopaedics.

[8] Callaghan JJ, Brand RA, Pedersen DR (1985). Hip arthrodesis. A long-term follow-up. The Journal of bone joint surgery American volume.

[9] Roberts CS, Fetto JF (1990). Functional outcome of hip fusion in the young patient. Follow-up study of 10 patients. J Arthroplast.

[10] Sponseller PD, McBeath AA, Perpich M (1984). Hip arthrodesis in young patients. A long-term follow-up study. The Journal of bone joint surgery American volume.

[11] Ding L, Gao YH, Li YR, Liu JG, Li SQ, Qi X (2018). Determinants of satisfaction following total hip arthroplasty in patients with ankylosing spondylitis. International orthopaedics.

[12] Kim YL, Shin SI, Nam KW, Yoo JJ, Kim YM, Kim HJ (2007). Total hip arthroplasty for bilaterally ankylosed hips. J Arthroplast.

[13] Kim YH, Oh SH, Kim JS, Lee SH. Total hip arthroplasty for the treatment of osseous ankylosed hips. Clinical orthopaedics and related research. 2003(414):136–48. 10.1097/01.blo.0000081935.75404.7f 12966287

[14] Feng DX, Zhang K, Zhang YM, Nian YW, Zhang J, Kang XM (2016). Bilaterally Primary Cementless Total Hip Arthroplasty for Severe Hip Ankylosis with Ankylosing Spondylitis. Orthopaedic surgery.

[15] Vosse D, de Vlam K (2009). Osteoporosis in rheumatoid arthritis and ankylosing spondylitis. Clin Exp Rheumatol.

[16] Kilgus DJ, Amstutz HC, Wolgin MA, Dorey FJ (1990). Joint replacement for ankylosed hips. The Journal of bone joint surgery American volume.

[17] Harris WH (1969). Traumatic arthritis of the hip after dislocation and acetabular fractures: treatment by mold arthroplasty. An end-result study using a new method of result evaluation. The Journal of bone joint surgery American volume.

[18] Ellapparadja P, Mahajan V, Deakin AH, Deep K (2015). Reproduction of Hip Offset and Leg Length in Navigated Total Hip Arthroplasty: How Accurate Are We?. J Arthroplast.

[19] Keršič M, Dolinar D, Antolič V, Mavčič B (2014). The impact of leg length discrepancy on clinical outcome of total hip arthroplasty: comparison of four measurement methods. J Arthroplast.

[20] Brooker AF, Bowerman JW, Robinson RA, Riley LH (1973). Jr. Ectopic ossification following total hip replacement. Incidence and a method of classification. The Journal of bone joint surgery American volume.

[21] Joshi RP, Eftekhar NS, McMahon DJ, Nercessian OA (1998). Osteolysis after Charnley primary low-friction arthroplasty. A comparison of two matched paired groups. The Journal of bone joint surgery British volume.

[22] Maloney WJ, Jasty M, Harris WH, Galante JO, Callaghan JJ (1990). Endosteal erosion in association with stable uncemented femoral components. The Journal of bone joint surgery American volume.

[23] DeLee JG, Charnley J. Radiological demarcation of cemented sockets in total hip replacement. Clinical orthopaedics and related research. 1976(121):20–32. 991504

[24] Engh CA, Massin P, Suthers KE. Roentgenographic assessment of the biologic fixation of porous-surfaced femoral components. Clinical orthopaedics and related research. 1990(257):107–28. 2199114

[25] Sodhi N, Mont MA (2019). Survival of total hip replacements. Lancet.

[26] Ye C, Liu R, Sun C, Lin J, Li H, Re H (2014). Cementless bilateral synchronous total hip arthroplasty in ankylosing spondylitis with hip ankylosis. International orthopaedics.

[27] Gautam D, Malhotra R (2019). Total Hip Arthroplasty in Ankylosing Spondylitis With Extension Contracture of Hips. J Arthroplast.

[28] Guo HZ, Yang CX, Tang ZP, Wang CX (2019). The effects of total hip arthroplasty in treating hip bony fusion in young and middle-aged patients with ankylosing spondylitis. J Orthop Surg Res.

[29] Khan T, Knowles D (2007). Damage to the superior gluteal nerve during the direct lateral approach to the hip: a cadaveric study. J Arthroplast.

[30] Ramesh M, O’Byrne JM, McCarthy N, Jarvis A, Mahalingham K, Cashman WF (1996). Damage to the superior gluteal nerve after the Hardinge approach to the hip. The Journal of bone joint surgery British volume.

[31] Zink A, Braun J, Listing J, Wollenhaupt J (2000). Disability and handicap in rheumatoid arthritis and ankylosing spondylitis–results from the German rheumatological database. German Collaborative Arthritis Centers. J Rhuematol.

[32] Benjamin M, McGonagle D (2001). The anatomical basis for disease localisation in seronegative spondyloarthropathy at entheses and related sites. Journal of anatomy.

[33] Idulhaq M, Park KS, Diwanji SR, Yoon TR, Wie JS (2010). Total hip arthroplasty for treatment of fused hip with 90 degrees flexion deformity. J Arthroplast.

[34] Pradhan R (1999). Planar anteversion of the acetabular cup as determined from plain anteroposterior radiographs. The Journal of bone joint surgery British volume.

[35] Morcos MW, Hart A, Antoniou J, Huk OL, Zukor DJ, Bergeron SG (2018). No Difference in Major Complication and Readmission Rates Following Simultaneous Bilateral vs Unilateral Total Hip Arthroplasty. J Arthroplast.

[36] Wang D, Wang HY, Luo ZY, Pei FX, Zhou ZK, Zeng WN (2019). Finding the Optimal Regimen for Oral Tranexamic Acid Administration in Primary Total Hip Arthroplasty: A Randomized Controlled Trial. The Journal of bone joint surgery American volume.

[37] Luo ZY, Wang HY, Wang D, Zhou K, Pei FX, Zhou ZK (2018). Oral vs Intravenous vs Topical Tranexamic Acid in Primary Hip Arthroplasty: A Prospective, Randomized, Double-Blind, Controlled Study. J Arthroplast.

[38] Blizzard DJ, Penrose CT, Sheets CZ, Seyler TM, Bolognesi MP, Brown CR (2017). Ankylosing Spondylitis Increases Perioperative and Postoperative Complications After Total Hip Arthroplasty. J Arthroplast.

[39] Kim YH, Kim JS, Cho SH (1999). Primary total hip arthroplasty with a cementless porous-coated anatomic total hip prosthesis: 10- to 12-year results of prospective and consecutive series. J Arthroplast.

[40] Wang W, Huang G, Huang T, Wu R (2014). Bilaterally primary cementless total hip arthroplasty in patients with ankylosing spondylitis. BMC Musculoskelet Disord.

[41] Zheng GQ, Zhang YG, Chen JY, Wang Y (2014). Decision making regarding spinal osteotomy and total hip replacement for ankylosing spondylitis: experience with 28 patients. The bone joint journal.

[42] Blizzard DJ, Nickel BT, Seyler TM, Bolognesi MP (2016). The Impact of Lumbar Spine Disease and Deformity on Total Hip Arthroplasty Outcomes. The Orthopedic clinics of North America.

